# Supplementary material for: Peridomestic natural breeding sites of Nyssomyia whitmani (Antunes and Coutinho) in an endemic area of tegumentary leishmaniasis in northeastern Argentina
Source: PLoS Negl Trop Dis. 2021 Aug 19;15(8):e0009676. doi: 10.1371/journal.pntd.0009676 (PMC8376073; doi:10.1371/journal.pntd.0009676)
Supplement: Attachment — Submitted filename: Letter to editor and responses to reviewer.docx [file pntd.0009676.s002.docx]

June 5, 2020

Paul Andrew Bates

Associate Editor
PLOS Neglected Tropical Disease

Ref.: Manuscript (PNTD-D-21-00480)

Dear Editor,

Please find enclosed the revised version of the manuscript PNTD-D-21-00480, entitled "Peridomestic natural breeding sites of *Nyssomyia whitmani* (Antunes and Coutinho) in an endemic area of cutaneous leishmaniasis in northeastern Argentina" by Mariana Manteca-Acosta, Regino Cavia, María Eugenia Utgés, Oscar Daniel Salomón, María Soledad Santini to be considered for publication in PLOS Neglected Tropical Diseases.

We greatly appreciate the useful comments and suggestions made by the reviewers to a previous version of our manuscript. After carefully considering all their recommendations, this new version has been thoroughly revised and corrected accordingly.

Point-by-point responses:

**METHODS**

Reviewer #3:

**Lines 114 to 115. ‘in the warmest month (January), and 7°C and 15°C in the coldest month (July), respectively” how the 15c coldest month? How about the humidity of the region?
Answer**. We inserted “The climate is subtropical without dry season; mean minimum and maximum temperatures, and relative humidity are 20°C, 30°C and 78% respectively in the warmest month (January); and 10°C, 22°C and 83% respectively in the coldest month (July) (taking the 1981-2010 period) between lines 117-120

**Line 120. Each of the seasons was used the same number of traps was used.
Answer**. Yes, in each season the number of traps was not modified. This information is in table 2 and 3.

**Line 128. Please add the manufacturer's detail PVC pipe tube.**

**Answer.** We inserted the manufacturer of the PVC pipe tube: SICA S.A, after “PVC” in line 130.

**Line 134. If trap was checked every 15-20 nights, how author would say “newly recorded as a newly emerged individual”. Also, as the author suggested keeping 15 -20 might cause deterioration of the fly, does this itself might cause repellent or attract the fly? Any prediction? Answer.** This is an accurate comment. We checked the trap every 15-20 days, for logistical and experimental design reasons we could not do it more frequently, and due to adhesive paper deterioration (it did not last 40 days), fungi and exposure to weather conditions it was not advisable to leave it longer. Therefore, as a compromise situation we selected the period of 15-20 days, to make this issue clearer in the text we changed the phrase “newly recorded as a newly emerged individual” to "new adults that emerged in the period of 15-20 days between trap revisions as newly emerged individual" between lines 141 and 143.

As an emergence trap we do not have any evidence or to generate hypotheses of repellency or attractiveness for larvae that pupate and emerge as adults.

**Line 147 to 149.** **Was this done at the same locations?**

Answer. This information is related between lines 168 and 183. The location of the first collection was farm 5 and the locations of the second collection were in eight farms. All farms are located in “Dos mil Hectareas”.

**Lines 187 to 190. I suggest using the COI amplification and sequencing methods to identify the species of the fly.**

**Answer.** Barcoding-COI is being done with the species in the area, for identification of both adults and larvae, but in this case the procedure is incompatible with the experimental design, as the phlebotomines remain inside the emergent trap for 15-20 days.

**The authors stated that “physicochemical characteristics (Table 1)” I don’t see any data on the table.**

**Answer.** The soil physicochemical characteristics are at the end of table 1: Humidity, pH, Total organic matter, Organic matter, Organic carbon, Total nitrogen, Carbon/nitrogen.

**References need any additional additions, particularly many of the scientific names were not italicized.**

**Answer.** Thank you. We have already edited the references.

**RESULT**

**Línea 454- could be their source or/and refuge area.**

**Answer.** We inserted “being a refuge area or an..” between lines 464 and 465.

Reviewer #1:

**- In the item Emergence trap: I am curious to know why only one trap size was used. I believe that, using the same collection effort, larger traps (with a larger diameter) would increase the sampled area, ok? If possible, I think it would be interesting to present an image of the emergency traps used and, preferably, fixed in any of the investigated environments.**

**Answer.** Only one size was used to avoid "trap diameter" being an additional factor of variability in the analysis. The trap size selected resulted from a compromise between feasibility of available trapping space in the different environments, and probability of contact of the emerging adult with the trap surface (adhesive paper), which decreases as the trap diameter increases.

Trap pictures are now included in the manuscript (New Figure 1). Pictures included the general aspect of the trap, the location on the field and an image showing a new emergent adult sticked in the adhesive paper	.

**- In the last line of page 5 - I believe that the collection effort is better defined as the number of traps and-or the sampled area, regardless of whether or not new emerged sand flies are captured.**

**Answer.** Thank you for your careful reading, here there was a mistake, we changed “The capture effort was estimated as the number of newly emerged phlebotomines captured per trap/nights. “to “The captured effort was estimated as the number of trap-nights” between lines 152 and 153.

**-In Results: “The period of greatest abundance of newly emerged phlebotomines was from October to January 2014, with a…” add the years: October 2014 to January 2015, ok?**

**Answer.** Ok. We inserted “October 2014 to January 2015“in the text in line 300.

**- Tale 1 and 2, draws attention to the difference in the values of “Total period productivity” for Chicken shed from Farm 5, as both were sampled in both periods and the capture of emerged sand flies was practically the same with the use of a same number of emergency traps (96). Review, okay?**

**Answer.** Looking at the increase in “total period productivity” from the first to the second collection we have the hypothesis that it increased in Farm 5 because the farmer improved the chicken shed construction.

**In addition, I believe that it would facilitate the understanding of these estimates to include information on the size (m2) of each** **environment in each Farm.**

**Answer**. Ok. We inserted this information in table 2 and 3.

**- In Discussion: In Brazil it is very common for chickens to be loose in the peridomicile, where they sleep on top of trees. Do you have this situation in any farm? and did you get to sample any soil under these trees?**

**Answer.** Yes, in our study area as well there are farms where the chickens sleep on top of trees. But, in this study, we selected only farms with chicken sheds since this environment is more common.

However, in other study, we sampled under these trees where the chickens sleep, and we captured sandflies as well. But these data are not published.

**- Have other insects, possibly predators of the immature forms of sand flies, been collected in the emergence traps?**

**Answer.** No, we did not find other insects.

**- regarding possible associations between the newly emerged in emergence trap and adults captured with light traps population, I would like to raise some questions for discussion with the authors; is there any information on latency (or diapause) of any phase of the sand flies life cycle for sub-tropical and tropical regions? Is it possible for males and females to survive for more than 30 days? Marking-release-recapture studies do not show this, okay? Possible associations between abundance of emerged in emergence trap and adults captured with light traps with a time difference greater than 2 months, it seems to me to be more influenced by climatic and sampling factors, on both populations, which are difficult to count. it is likely that the climatic conditions on the days of the collections, or on the previous days, influence more collections of adult forms than immature forms.**

**Answer.** Evidence on latency, diapause or survival of American subtropical phlebotomine in general and of the species of interest in particular is scarce, even in controlled experiments or by inferences based on several estimated parameters. Although effectively capture-recapture studies suggest a relatively short ‘mode’ of adult survival, captures in short warm episodes during the cooler season demonstrate the possibility of emergence of developing larvae, as well as the permanence of adults in places with more stable climatic conditions (chicken coops) can alter the seasonal pattern. We agree that the climatic conditions of the day of capture affect especially the adults because they modulate the activity (metabolism and partial vapor pressure of attracting plumes), while climatic conditions with time lags affect the population size in successive incremental or decremental cohorts according to the area suitable for oviposition and breadinf, developmental success-any stage-, reproduction, etc.

**- an issue that always leaves a doubt for researchers trying to discover the preferred breeding sites of sand flies is the uncertainty about whether the number of samples in each investigated environment was sufficient to determine their favorable or unfavorable condition to serve as a breeding site. Not to mention the existence of other possible (and unknown) sites. Did the authors also get this feeling?**

**Answer.** Yes, we had the same doubts. Which is the number of sandflies sufficient to consider an environment as a breeding site? Will they be distributed throughout the peridomicile, or will they be aggregated in the environments? Due to the difficulty of finding them, many times those of us who carry out these studies settle for a few numbers of individuals (which also possibly varies between species).

However, while there may be other potential breeding environments for sandflies in the study area, this work shows that among the most productive environments are the chicken shed and below house since the number of emergents that we estimate to have emerged may explain the abundance of adults occurring in these farms.

We hope that this revised manuscript is now suitable for publication in PLOS Neglected Tropical Diseases.

Sincerely yours,

Dra. Mariana Manteca Acosta
